# Effects of forest degradation classification on the uncertainty of aboveground carbon estimates in the Amazon

**DOI:** 10.1186/s13021-023-00221-5

**Published:** 2023-02-14

**Authors:** Ekena Rangel Pinagé, Michael Keller, Christopher P. Peck, Marcos Longo, Paul Duffy, Ovidiu Csillik

**Affiliations:** 1 College of Forestry, Oregon State University, Corvallis, OR 97333 USA; 2 International Institute of Tropical Forestry, USDA Forest Service, Río Piedras, 00926 Puerto Rico; 3 Jet Propulsion Laboratory, California Institute of Technology, Pasadena, CA 91109 USA; 4 Neptune and Company, Inc, Lakewood, CO 80215 USA; 5 Climate and Ecosystem Sciences Division, Lawrence Berkeley National Laboratory, Berkeley, CA 94720 USA

**Keywords:** Forest degradation, Selective logging, Forest fire, Very high-resolution imagery, Probabilistic classification, Airborne lidar, Biomass, Amazon

## Abstract

**Background:**

Tropical forests are critical for the global carbon budget, yet they have been threatened by deforestation and forest degradation by fire, selective logging, and fragmentation. Existing uncertainties on land cover classification and in biomass estimates hinder accurate attribution of carbon emissions to specific forest classes. In this study, we used textural metrics derived from PlanetScope images to implement a probabilistic classification framework to identify intact, logged and burned forests in three Amazonian sites. We also estimated biomass for these forest classes using airborne lidar and compared biomass uncertainties using the lidar-derived estimates only to biomass uncertainties considering the forest degradation classification as well.

**Results:**

Our classification approach reached overall accuracy of 0.86, with accuracy at individual sites varying from 0.69 to 0.93. Logged forests showed variable biomass changes, while burned forests showed an average carbon loss of 35%. We found that including uncertainty in forest degradation classification significantly increased uncertainty and decreased estimates of mean carbon density in two of the three test sites.

**Conclusions:**

Our findings indicate that the attribution of biomass changes to forest degradation classes needs to account for the uncertainty in forest degradation classification. By combining very high-resolution images with lidar data, we could attribute carbon stock changes to specific pathways of forest degradation. This approach also allows quantifying uncertainties of carbon emissions associated with forest degradation through logging and fire. Both the attribution and uncertainty quantification provide critical information for national greenhouse gas inventories.

## Background

Tropical forests account for more than half of terrestrial aboveground biomass carbon stocks, and host 60–70% of terrestrial species, despite covering only 7–10% of the land [1–3]. Deforestation, selective logging, fires, and fragmentation have greatly altered forests in recent decades across the tropics [4–6]. Second growth and degraded forests cover more area today than intact forests, although the full extent of tropical forest degradation is highly uncertain [7–11]. Likewise, carbon losses attributed to degradation may be similar to or exceed deforestation-related losses [12–20]. The uncertainty for this carbon source is partly due to the aforementioned uncertainty in degradation area but is also due to the variability in carbon stocks in degraded forests at different spatial and temporal scales [14, 21–27]. The consequences of forest degradation for potential future losses and gains (through regeneration) for carbon budgets, forest structure and biodiversity remain largely unknown [28–31].

Since the late 1990s, researchers have used moderate resolution remote sensing for detection of logging and fire effects on forests in Amazonia [10, 32–35]. Linear spectral mixture modelling using passive optical data has been the main tool for this approach [10, 36–38]. Manual auditing has been required in regional applications [8, 39, 40]. More recent studies employ a combination of spectral and textural information with machine learning [41, 42]. The addition of temporal filtering to spectral data permits detection of understory forest fires at varied resolutions when adequate temporal information is available [43]. Recent work identifying forest degradation in the Amazon using Landsat time series is promising [7, 44] but does not separate the main degradation drivers (e.g., logging vs. fire) or estimate the effects of degradation on biomass. Textural analysis of very high resolution (VHR) images has been applied to local studies of logging and forest structural characteristics, although the cost of acquiring and processing data over large areas has remained a challenge [45–47]. The advent of high frequency and more accessible VHR images such as the PlanetScope archive, along with increased processing capabilities, has opened new possibilities for detailed forest degradation detection and monitoring (e.g., [48]).

Deforestation and forest degradation in the Brazilian Amazon are both substantial sources of carbon to the atmosphere [30, 49, 50]. Biomass stocks have been quantified through remote sensing data calibrated with field inventory data [51–54] although at a fine spatial scale, changes in forest condition are highly uncertain. Uncertainty arises from the inconsistency of land cover maps and the variability in land change area estimates, which can have dramatic effects on the outputs of models that rely on such information [55]. Biomass estimates from current and near-future space missions (e.g., NASA GEDI and ESA BIOMASS) will greatly reduce uncertainties at the 1 km^2^ resolution [56–58] but these missions will only provide a view of forest biomass over a relatively brief period [3 to 5 years]. While GEDI presents a spectacular advance in our knowledge of biomass and other aspects of forest structure, it is a sampling mission that will inevitably leave spatial gaps. Airborne lidar has provided high spatial resolution and accurate aboveground biomass information [26, 59] for intact and degraded forest but its coverage is sparse.

Effective management of carbon dynamics related to forest land use change requires accurate attribution of carbon sources and sinks. As noted above, forest degradation is an important and poorly quantified carbon source while recovery after degradation is an overlooked potential carbon sink. The promotion of forest carbon sinks is a potentially cost-effective approach for mitigating climate change within an overall strategy of forest carbon management and conservation [61, 62]. Ultimately, improving the ability to accurately depict biomass changes associated with degradation requires knowledge of where and when forests were degraded, the biomass of degraded versus intact forests, and the quantification of estimated uncertainties.

Classification of tropical forest degradation and associated biomass estimation remain a significant challenge because existing uncertainties in this type of classification and in biomass estimation hinder accurate attribution of carbon emissions to specific forest classes. In this study, we classify intact and degraded forests and combine degradation probability estimates with biomass estimates. We use textural features from commercial very high-resolution optical data to develop a probabilistic classification of intact, logged, and burned forests at selected sites in the Brazilian Amazon. We combine our forest classification with aboveground biomass distributions derived from airborne lidar data using a Monte Carlo simulation and compare biomass uncertainties from lidar data only to uncertainties estimated when both the uncertainty of forest degradation classification and biomass uncertainty are considered. We address the following research questions:

- What is the accuracy of a forest degradation classification using a machine learning approach?
- What is the impact of forest degradation on biomass estimates?
- How does the uncertainty of forest degradation classification affect biomass estimates and uncertainty in degraded forests?

## Methods

**Fig. 1:**
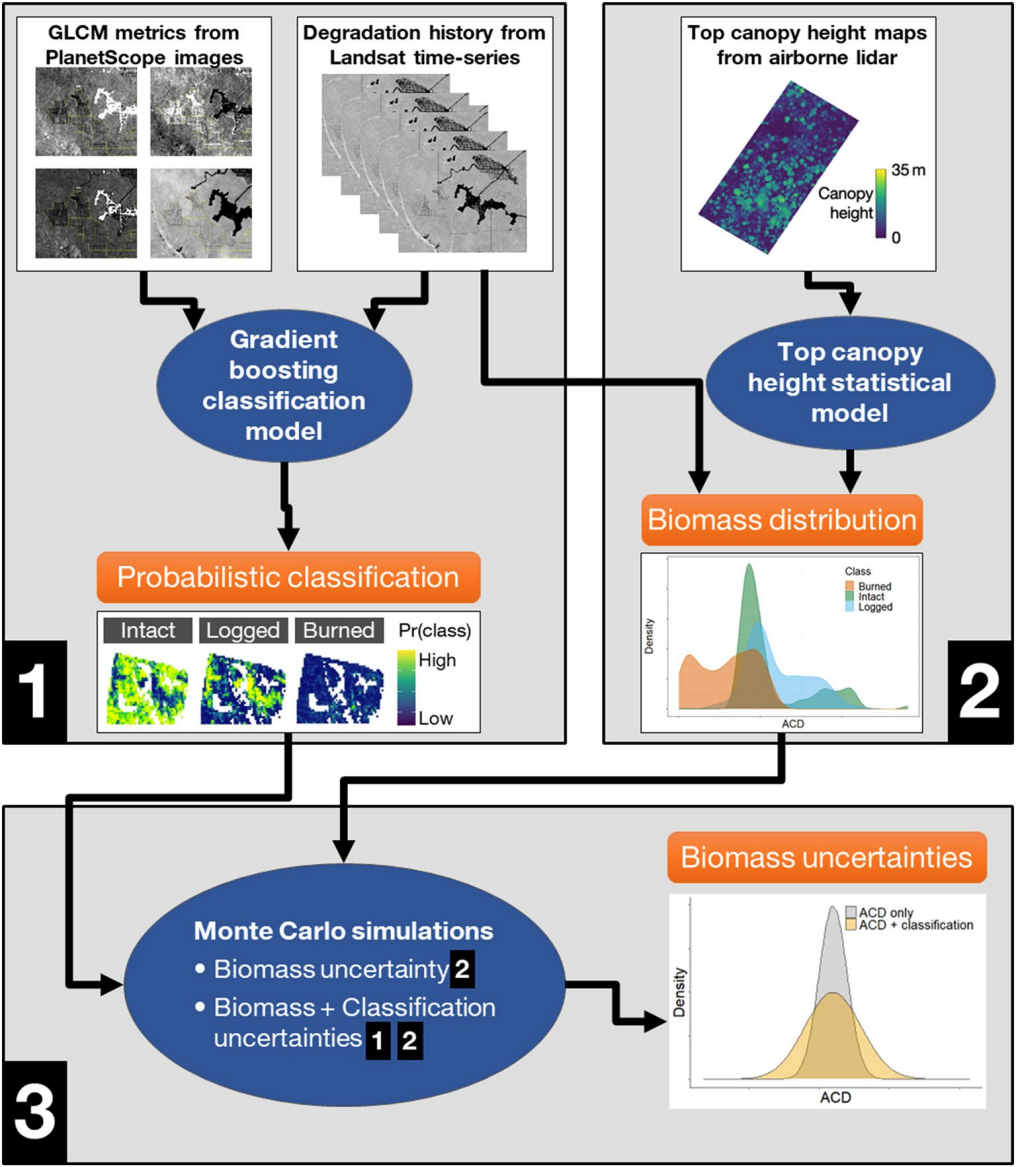
Diagram of the steps of our study including (**1**) probabilistic classification of Planet VHR images, (**2**) biomass estimation from airborne lidar, and (**3**) Monte Carlos simulations to estimate landscape level above ground biomass and uncertainties related to both biomass estimation and forest degradation classification

To address research question (a), we implemented a gradient boosted classification modelling framework with forest degradation history from Landsat time-series as reference data and textural metrics derived from PlanetScope images as predictors (Fig. 1, box 1). To address research question (b), we estimated biomass distributions of intact, logged and burned forests from airborne lidar data (Fig. 1, box 2). To address question (c), we compared biomass estimates from a Monte Carlo simulation that accounted for biomass uncertainty from lidar data to a simulation that also accounted for forest degradation classification uncertainty from the model referenced in (a) (Fig. 1, box 3).

### Study sites

**Fig. 2:**
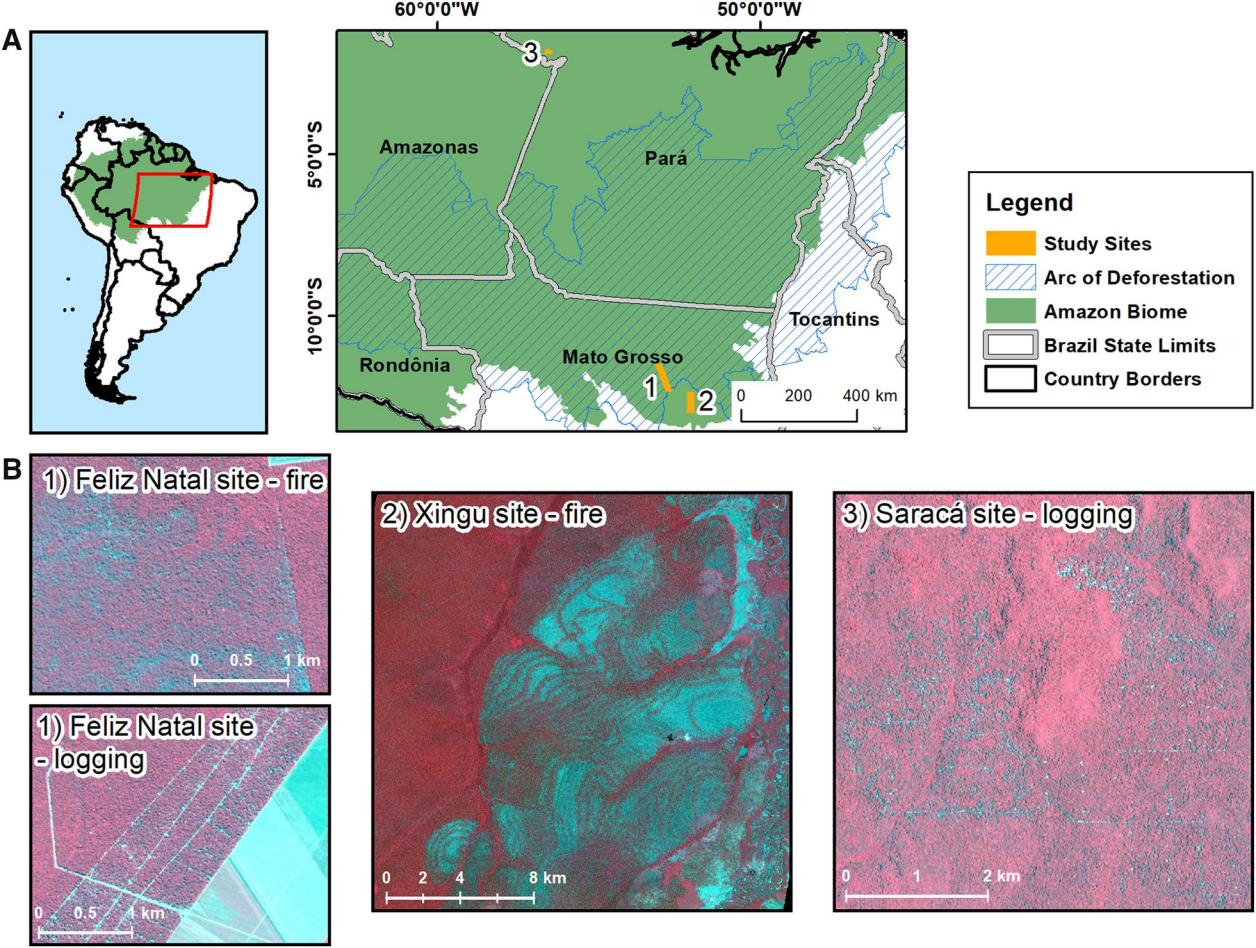
Location of the study sites in the Amazon (**A**). The insets (**B**) show forest degradation from logging and fire on PlanetScope false color composites (near-infrared, red, green) for the three sites

We conducted our study at three sites in the Brazilian Amazon that covered portions of the Feliz Natal Municipality and Xingu Indigenous Territory in the Brazilian state of Mato Grosso, and Saracá-Taquera National Forest, in the Brazilian state of Pará (Fig. 2). The Feliz Natal site comprises a mixture of land covers, whereas Xingu and Saracá sites were included as mixtures of intact forest with logging and fires only, respectively.

Feliz Natal (Fig. 2, site 1) is located in the Brazilian Arc of Deforestation, a government-defined region that concentrates 70% of deforestation within 100 municipalities (~ 1 million km^2^). Feliz Natal has a diversity of land uses including pastures and mechanized agriculture that have replaced part of the original forest cover. Most of the remaining forests in this region have been logged and a substantial portion of the forests have burned at least once [26].

The Xingu site (Fig. 2, site 2) lies about 80 km southeast from the Feliz Natal site, but it is located within the Xingu Indigenous Territory. Indigenous lands are well-known barriers for deforestation and other anthropogenic pressures in the Amazon [63, 64], however large fires occurred in 2016 and 2017 in the Xingu area [65]. The climate of Feliz Natal and Xingu region is typical of south-eastern Amazonia, with mean annual precipitation of about 1900 mm, an extended 5-month dry season and mean annual temperature of 25 °C [66].

The Saracá-Taquera National Forest (Fig. 2, site 3) holds a federal logging concession administered by the Brazilian Forest Service. Along with a long history of bauxite mining in this National Forest, specific areas were assigned for sustainable forest management. Selective logging at this site was conducted between 2015 and 2020 using reduced-impact logging techniques [67] to comply with federal regulations. The climate at this site is tropical humid, with mean annual precipitation of 2000 mm, and dry season extending from July to October [68].

### Classification of forest degradation

#### Reference data for forest degradation classification

Our forest degradation reference dataset was built from visual interpretation of Landsat TM, ETM + and OLI time-series, from 1984 to 2020. At least one cloud-free image per year was available for each site. For each image of the time-series, we computed the Normalized Burn Ratio (NBR), a spectral index that has been widely used to detect forest disturbances [69, 70]. We then manually delineated fire and selective logging polygons and recorded the year of the degradation event. We masked out water and wetlands based on Gumbricht, Roman-Cuesta [71], and deforestation using the Brazilian PRODES classification [72].

#### PlanetScope images

We selected one multispectral PlanetScope image with four spectral bands (blue, green, red, and near infrared) [73] for each site. For each image we calculated the Enhanced Vegetation Index (EVI, [74]), to highlight both recent degradation and subsequent regeneration. Image dates were selected based on the disturbance occurrence on each site and proportion of cloud cover (Table 1).

**Table 1:** Overview of PlanetScope and lidar data for each site

Site	PlanetScope data		Airborne lidar data			
	Image date	Area (ha)	Year(s) of acquisition	Area of intact forests (ha)	Area of logged forests (ha)	Area of burned forests (ha)
Feliz Natal	17-Jun-2018	235,872	2017–2018	717	1574	1751
Xingu	18-Oct-2017	191,823	2017	740	0	554
Saracá	19-Sep-2020	45,756	2013–2015	855	2082	0
Total		473,451		2312	3656	2305

#### The GLCM approach and generation of predictors

We used the Gray-Level Co-Occurrence Matrix (GLCM) textural technique [75] to calculate metrics used to classify degraded forests in our test sites. Texture in images quantifies pixel grey level differences, size of area where change occurs (neighbourhood, defined by a window size), and directionality [76]. GLCM tabulates how often different combinations of pixel grey levels occur in each image and then derives statistics from this tabulation. The eight GLCM metrics used in this study can be categorized into three groups: (1) descriptive statistics, which include mean, variance, and correlation; (2) contrast, which includes contrast, homogeneity, and dissimilarity; (3) and orderliness or regularity, which includes angular second moment and entropy. Description of the GLCM metrics and practical guidelines for choosing GLCM metrics for classifying remote sensing images can be found in Hall-Beyer [77] and Hall-Beyer [76].

We generated the GLCM metrics for the Planet-derived EVI using the *glcm* package [78] in R [79]. After empirical tests, we selected the following parameters for the *glcm* function: window size of 45 pixels (140.625 m); and shift as the average across all directions (i.e., no effects of directionality in the observed phenomena). We trimmed the outermost window along the edge of each image to avoid artifacts where there was insufficient information for GLCM to compute accurate textural values.

Although the textural feature window size of about 141 m captures considerable heterogeneity associated with degraded forest patches, we explored aggregating windows to a coarser spatial resolution for more accurate classification [80]. We tested different aggregation resolutions (140.625 m, 281.25 m, 562.5 m, and 1125 m; corresponding to 45, 90, 180 and 360 PlanetScope pixels, respectively), and based on model performance, we selected the 562.5 m resolution. The following resampling statistics were used to aggregate the GLCM metrics from the native resolution (3.125 m) to 562.5 m grid cells: average, standard deviation, skewness, root mean square, minimum, first quartile, median, third quartile and maximum. In total, 72 raster layers (9 resampling statistics for each of the 8 GLCM metrics) were used as explanatory variables for the classification model.

#### Probabilistic classification model

Rather than selecting a single hard classification for each grid cell of our image, we quantified the probability that grid cells would fall into each of three classes: intact, logged, and burned (Fig. 1, box 1). Gradient boosted trees were used to classify grid-level degradation because of their strong predictive performance and flexibility in accommodating typical features of data such as nonlinearities and interactions [81]. Our multinomial classification tree model was fitted using the stochastic gradient boosting algorithm implemented in the *xgboost* R package [82]. The multi:softprob objective function was utilized to output a grid-level prediction containing an estimated probability of belonging to each degradation class.

Because degradation classes were unbalanced, a class-weighted loss function was utilized during model training. We specified a weight variable such that the sum of individual observation weights within each class was equal across the three degradation classes. Individual observation weights were subsequently multiplied by the grid cell associated purity (i.e., the percentage of the grid cell that is occupied by the dominant class) to down-weight error contributions from less homogeneous reference data. The full dataset was partitioned into training (50%) and test (50%) sets using stratified random sampling to balance class distributions within each split. The stratification variable consisted of binned purity values, in increments of 0.2, within each disturbance class. GLCM features were centered and scaled within each site to put each feature on a common scale after combining data for the three sites.

Several techniques were utilized to avoid overfitting during training and optimize the model’s bias-variance trade-off. First, we randomly selected (without replacement) 80% of the training data and 70% of the GLCM features to be utilized within each boosting round. This stochasticity decorrelates the decision trees and increases the predictive performance of the ensemble. Second, we used early stopping to halt training once the multiclass error rate for the validation set failed to decrease after five boosting iterations, thus rapidly detecting the inflection point of the learning curve. Finally, we set the learning rate to 0.2.

We determined optimal ranges for each hyperparameter through an iterative grid search. Terminal nodes were allowed to have a minimum of five observations, the maximum tree depth within a boosting round was equal to four, and a reduction of 0.2 in the multiclass error rate was required to further partition a leaf node. To assess model performance while accounting for uncertainty in both partition variability and algorithm stochasticity, we fitted classification models to 100 randomly generated partitions of the data.

Several studies showed that the degradation signal fades from optical images within 3–5 years [36, 83, 84] because of forest regeneration. We excluded from model training the grid cells with logging and fire disturbances that occurred more than five years prior to the date of the image, because preliminary model fits indicated increased confusion for older disturbances.

Adhering to good practice of accuracy assessments as suggested by Olofsson, Foody [55], we report the accuracy of our classification by presenting the confusion or error matrix and the most common accuracy measures. These assessments are presented for the highest predicted probability of forest degradation class for each grid cell as if we had conducted a *hard* as opposed to a probabilistic classification: *Overall accuracy*, which is simply the proportion of the area mapped correctly. It provides the user of the map with the probability that a randomly selected location on the map is correctly classified. *User's accuracy* is the proportion of the area mapped as a particular category that is actually in that category in the reference data. *Producer's accuracy* is the proportion of the area that is a particular category in the reference data that is also mapped as that category.

### Biomass estimates

To estimate grid cell-level biomass, we used high point-density lidar data collected over or adjacent to the test sites [85]. In total, 8723 hectares of airborne lidar over intact, logged, and burned forests were included (Table 1). When classifying the lidar transects over degraded forests, no distinction was made with regard to time since disturbance. Biomass was estimated as aboveground carbon density (ACD) from the lidar-derived top-of-canopy height at 50 m plot resolution (0.25 ha) using the methods detailed in Longo, Keller [60] and then resampled to 500 m resolution (25 ha) using the median values of the pixels in the 10 × 10 plot-equivalents, to approximately match the resolution of the classification predictors (Fig. 1, box 2). We reported the ACD estimates of Feliz Natal and Xingu sites together, because of their spatial proximity and similar ACD range.

### Estimation of uncertainties

Monte Carlo simulation was used to quantify uncertainty in grid-level aboveground carbon density estimates (Fig. 1, box 3). We performed simulations that accounted for uncertainty from two different sources: (i) ACD estimation and (ii) forest degradation classification. The first simulation accounted for both sources of uncertainty (labelled ACD + Classification) and the second simulation accounted for only uncertainty in ACD estimates (labelled ACD only). For each Monte Carlo iteration, individual grid cells were classified using the predicted class probabilities from the multinomial classification model. For the first simulation, cells were classified based on random sampling from the predicted probabilities and the second simulation classified cells by selecting the class with the highest predicted probability. ACD on a per-grid cell basis was then assigned by randomly sampling from the associated site and degradation class carbon density estimates. Site-level carbon density statistics were computed for a total of 10,000 iterations.

The patchiness of disturbed forests implies some degree of spatial correlation on the landscape. One of the useful features of the Monte Carlo approach for estimating uncertainty is that is rests upon few assumptions compared to complex geostatistical models, and provides an estimate of the uncertainty that is conditional on the complex spatial patterns of the landscape.

## Results

**Fig. 3:**
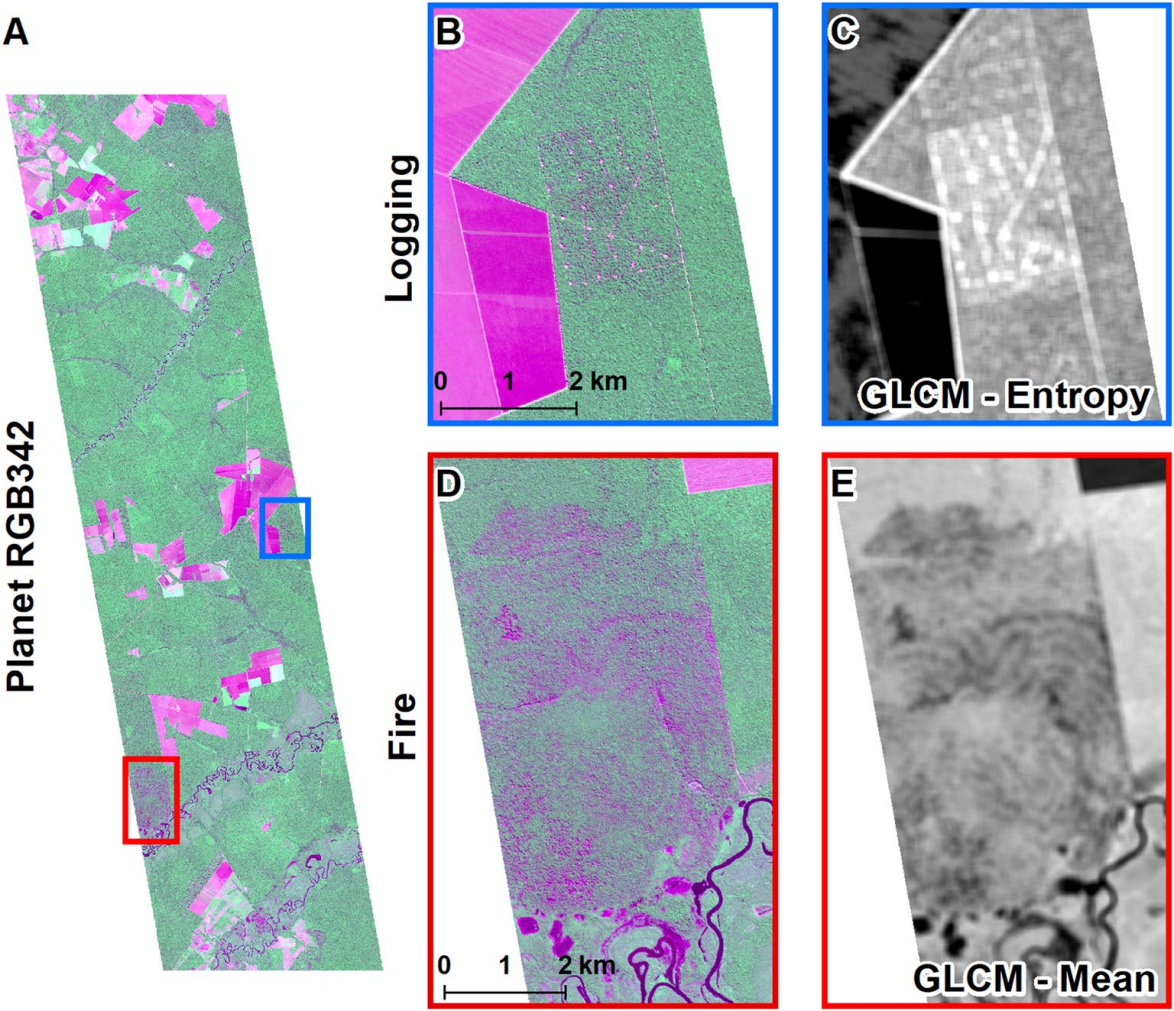
Feliz Natal test site: entire PlanetScope image false color composite (red, near-infrared, green) (**A**), insets showing logging and fire events (**B** and **D**), and selected GLCM metrics for the same area (**C** and **E**). The fire occurred in 2017, and logging in 2017 and 2018; PlanetScope image is from 2018

GLCM textural metrics responded to forest degradation, although different metrics were most effective to highlight logging and fires (Fig. 3). The entropy metric, which emphasizes contrasting edges, captured the roads and log storage decks in logging areas (Fig. 3C). The GLCM mean metric responded strongly to the diffuse changes visible in recently burned forests (Fig. 3E).

### Probabilistic classification of forest degradation

**Fig. 4:**
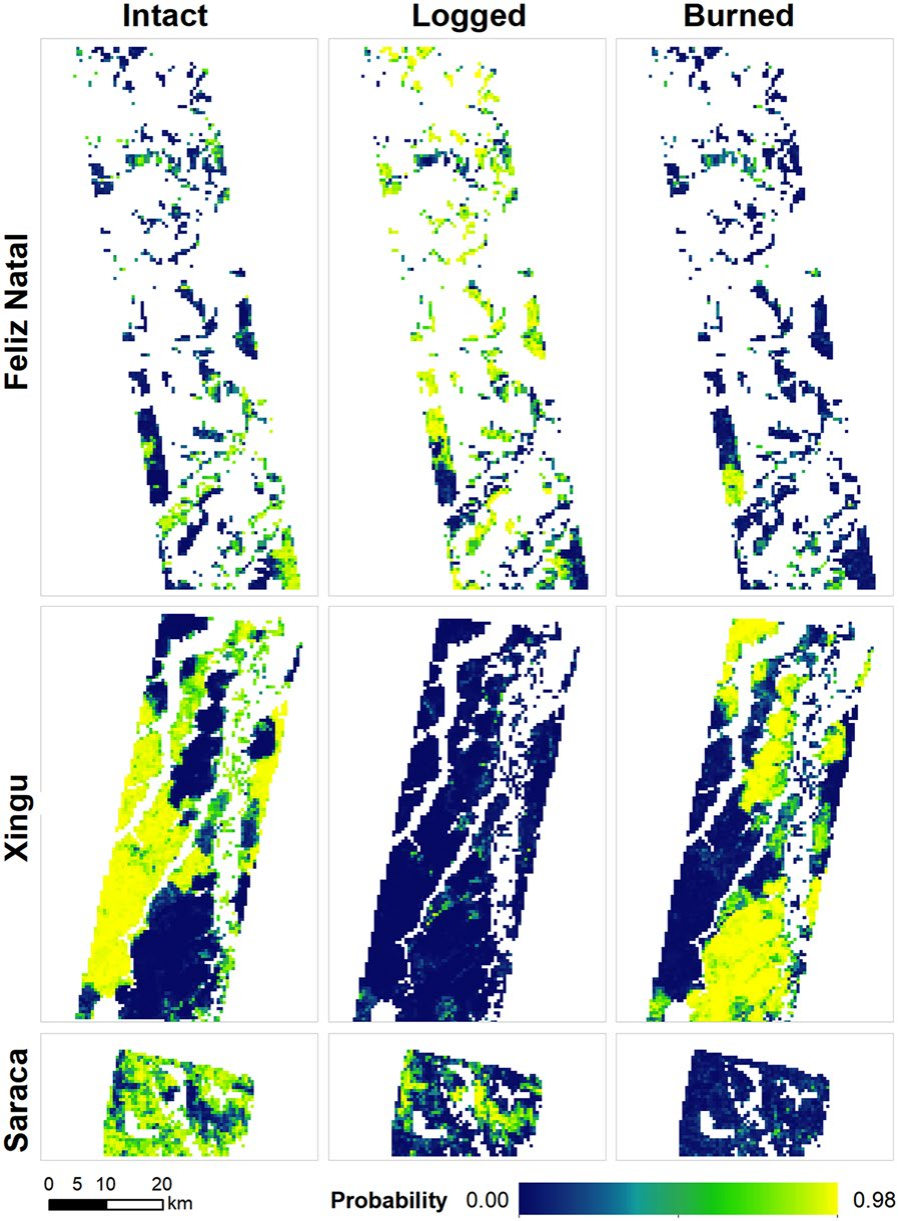
Probabilities of class occurrence for each site, as predicted by the multi-site model, at each 562.5 × 562.5-m grid cell. No-data grid cells within the areas represent either masked areas or forests degraded more than five years prior to image acquisition

We achieved high accuracy when we compared the most probable forest degradation class to reference data reserved for testing the model (Table 2, Fig. 4). The overall accuracy of the multi-site model (a single model including data from the 3 test sites) was 0.86 (95% CI 0.85–0.87), whereas the accuracies obtained for the individual sites were 0.69 (95% CI 0.65–0.72), 0.93 (95% CI 0.91–0.94) and 0.88 (95% CI 0.85–0.90) for Feliz Natal, Xingu and Saracá sites, respectively.

**Table 2:** Confusion matrix for the model using the 50% of the data held out for testing

	Class	Reference				Producer's accuracy	User’s accuracy	Overall accuracy
		Burned	Logged	Intact	Total			
Predicted	Burned	991	16	82	1089	0.88	0.91	0.86 ± 0.1
	Logged	26	366	194	586	0.85	0.62	
	Intact	107	47	1612	1766	0.85	0.91	
	Total	1124	429	1888	3441			

Predicted classes are the rows and the reference classes are the columns. The main diagonal denotes grid cells which have been classified correctly and the off-diagonal elements represent incorrect predictions

Producer’s and user’s accuracies for grid cells classified by the most probable degradation class were variable among classes. The burned forest class showed the highest producer’s and user’s accuracy (Table 2). In contrast, there was greater confusion between intact and logged forest classes because canopy damage caused by logging can sometimes be subtle to optical sensors, and because apparently intact forests may be degraded by fragmentation.

**Fig. 5:**
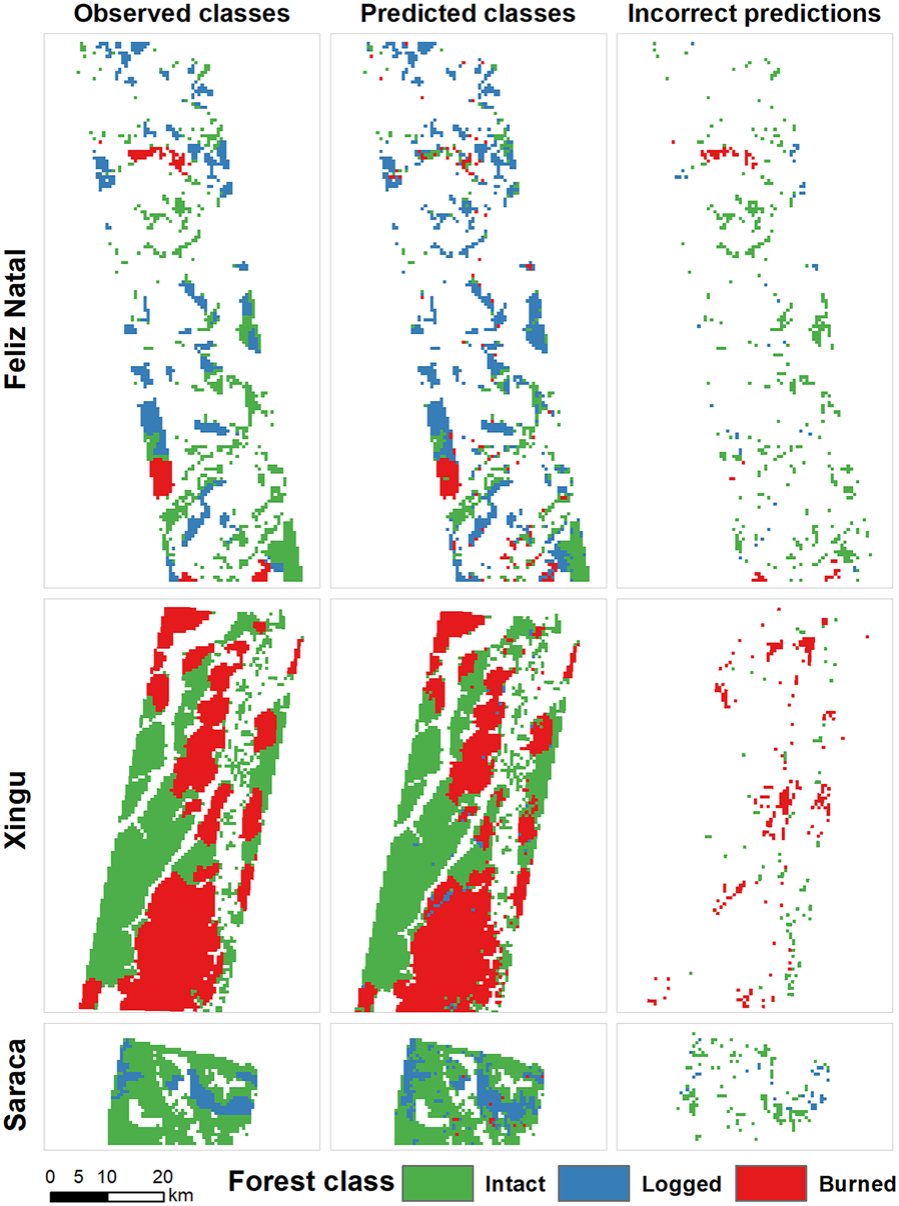
Observed (left panel) versus predicted (center panel) classes, and incorrect class prediction (right panel) for the 562.5-m grid cells. The rightmost panels present the reference (correct) classification for the incorrect predictions

Overall, we found a good match between the estimated classification and our reference data (Figs. 4 and 5). While our reference data do not include fire occurrences in the Saracá site, the fire class was selected for hard (most probable) classification in 1.2% of the grid cells. Similarly, there were no logging occurrences in the Xingu site in the last five years; however, the logging class was selected for hard classification in 1.1% of the grid cells at this site (Fig. 5).

### Biomass estimates for intact and degraded forests

**Fig. 6:**
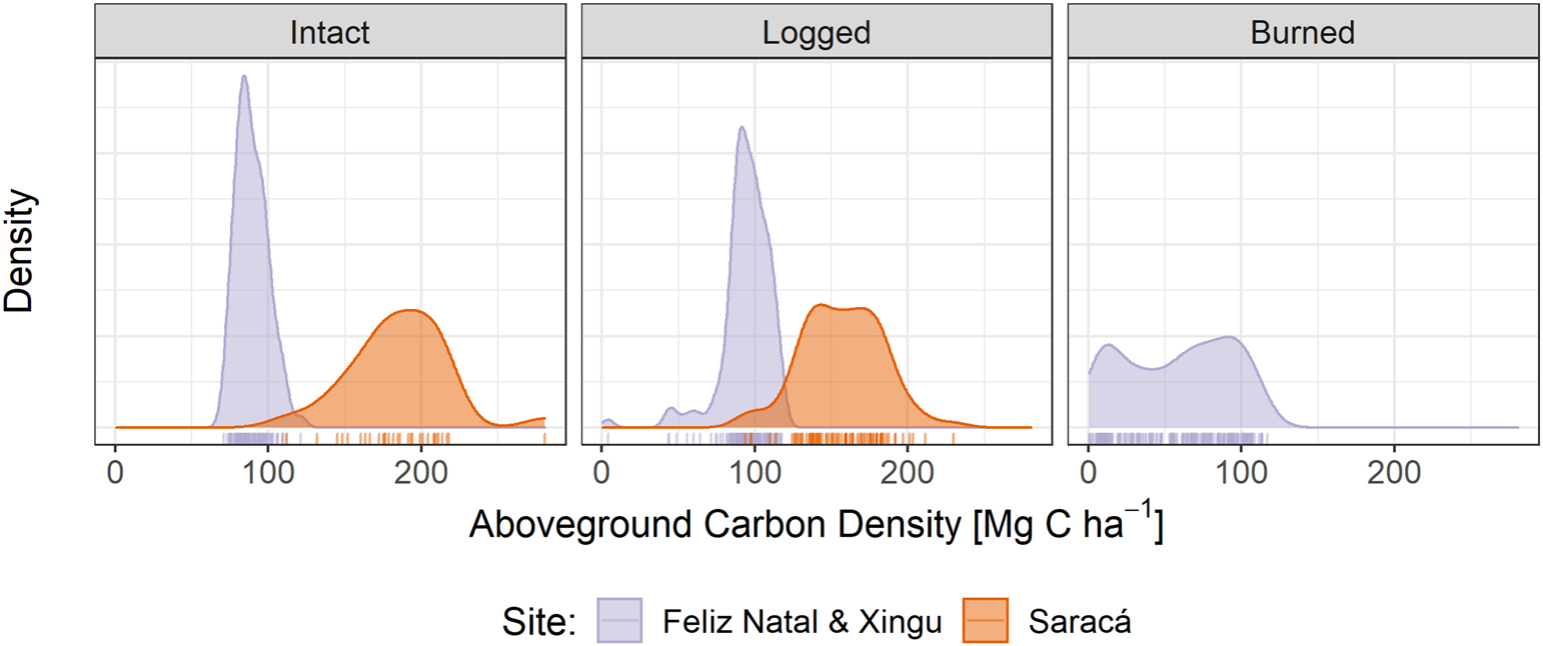
Probability density function of lidar-derived ACD for the degradation classes and sites. There were no burned areas at the Saracá site nor logged areas in the Xingu site in the 5 years prior to the image acquisition for those sites

The distributions of lidar-derived biomass estimates differed substantially among sites and degradation classes (Fig. 6, Table 3). The mean ACD for intact forests showed a two-fold difference between the combined Feliz Natal and Xingu sites versus Saracá. Interestingly, mean ACD is approximately 6% higher in logged forests than in intact forests in Feliz Natal and Xingu, but in Saracá, logging caused mean ACD reductions of approximately 15%. In the combined Feliz Natal and Xingu sites, we observed an average ACD decrease of 35% in burned forests compared to intact forests.

**Table 3:** ACD mean (and standard deviation) for each forest degradation class and site from lidar data and reference site classification

Class	Feliz Natal and Xingu	Saracá
Intact	89.2 (10.2)	185.3 (31.9)
Logged	93.9 (16.3)	156.8 (26.0)
Burned	57.9 (35.0)	–

ACD units are Mg C ha^−1^

### Combined uncertainties from biomass estimates and forest degradation classification

**Fig. 7:**
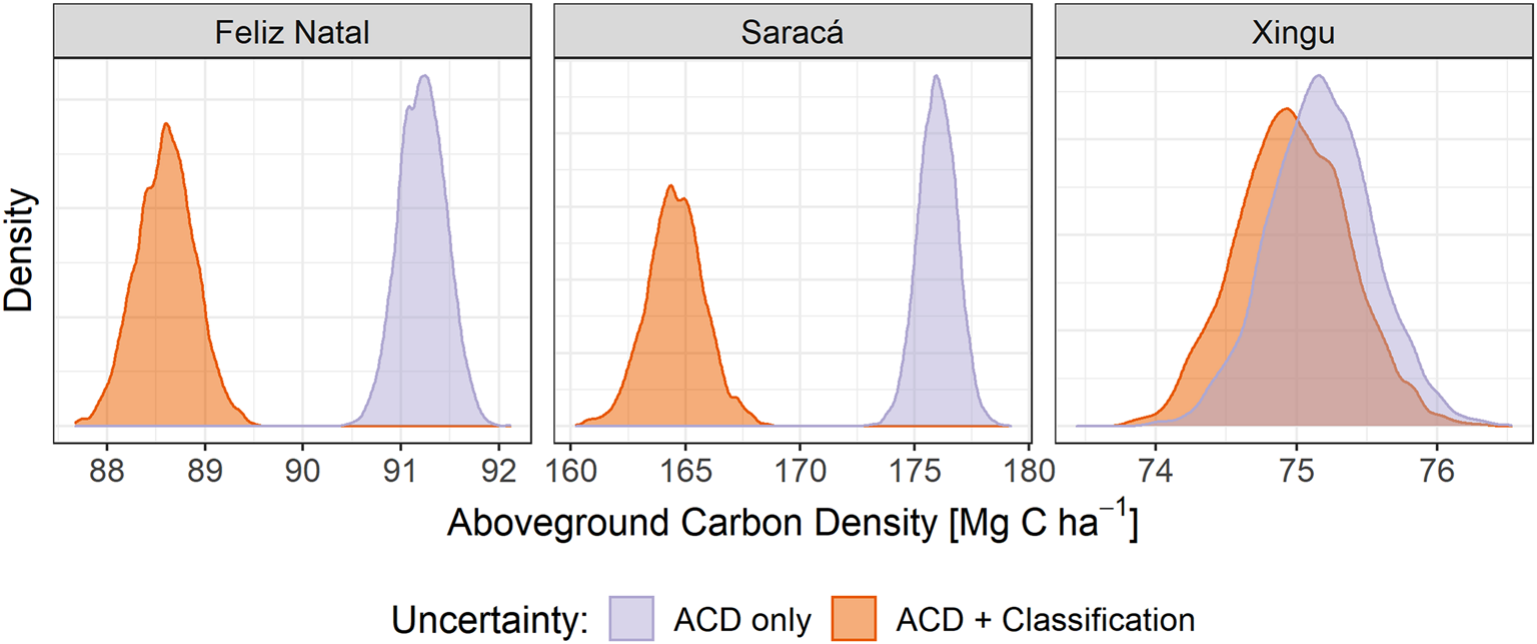
ACD distributions considering uncertainties from ACD estimates only, and both ACD and forest degradation classification uncertainties

We estimated ACD for the study area by combining the ACD distributions for each forest class with the probabilistic forest degradation classification using a Monte Carlo simulation. We expected that combining the uncertainties of classification and the uncertainties of ACD estimation would increase overall ACD uncertainty without substantially affecting means. Instead, we found that the uncertainties varied only slightly between the two approaches, but the mean ACD value was lower in all cases when the uncertainty of probabilistic classification was included (Fig. 7, Table 4). The uncertainty, as given by the standard deviation, was low in all cases, but always greater for ACD + Classification than for ACD only (Table 4). In Feliz Natal and Saracá sites, the ACD distributions from the two types of uncertainties did not even overlap, whereas distributions overlap considerably in the Xingu site.

**Table 4:** ACD mean (and standard deviation) for each test site and uncertainty approach

Uncertainty approach	Feliz Natal	Saracá	Xingu
ACD only	91.2 (0.23)	176.0 (0.82)	75.2 (0.38)
ACD + Classification	88.6 (0.30)	164.6 (1.26)	75.0 (0.40)

ACD units are Mg C ha^−1^

## Discussion

We presented an approach to classify tropical forest degradation based on VHR optical images and machine learning classification that achieved high accuracy on three distinct test sites. Generalizing this approach will require testing with reference data covering a wide range of terrain, forest types, and land-use characteristics. Nonetheless, our results represent an advance towards improved forest degradation classification in the Amazon. Combining improved classification and recent innovations in carbon stock estimation using satellite sensors, future studies can undoubtedly improve our understanding of the role of forest degradation in the Amazon regional and pantropical forest carbon budgets.

### Classification of degraded forests in the Amazon

In order to demonstrate the effectiveness of our probabilistic classification, we treated the most probable class as a discrete output (i.e., a hard classification). However, mixed pixels with multiple land covers are rather common in the Amazon and represent a challenge to our forest degradation classification [86]. The probabilistic classification allowed us to take this characteristic into account. The challenge of mixed pixels gains importance when classified grid cells are larger. Moreover, a probabilistic forest degradation classification allowed us to explicitly incorporate the classification uncertainty into landscape ACD estimates. We used the information on the probability of class membership to indicate the confidence associated with class allocation, indicating classification reliability [87].

While we satisfactorily classified recent disturbances with Planet data (< 5 years), the spectral signal of disturbance quickly fades due to the vegetation regrowth following fire and logging events [27, 36]. In contrast, structural changes (e.g. measured using airborne lidar) are much more persistent [80, 88]. To mitigate the temporal limitation imposed by forest regeneration, we imposed a 5-year disturbance age cut-off. Implementation of this approach for operational monitoring would require frequent updates, preferably in intervals shorter than 5 years. The advent of easily accessible high frequency VHR images such as those from the Norway's International Climate and Forests Initiative (NICFI) Satellite Data Program makes this task feasible on a yearly or even perhaps more frequent basis, even for high-cloud coverage regions such as tropical forests.

We did not take into consideration the effects of overlapping disturbances in the classification. In hotspots of land use change such as the Arc of Deforestation, the effects of repeated fire and logging occurrences on biomass are not independent [26]. For simplicity, we only used the most recent degradation event prior to the Planet image date to calibrate our classification model, but we acknowledge that disturbance history may affect forest degradation classification. In particular, forest fires may accentuate previous degradation patterns from logging when they preferentially burn small stature regenerating vegetation on logging roads and log storage decks. In addition, selecting only the most recent disturbance may be affecting the corresponding accuracy metrics (e.g., the model could be correctly classifying previous disturbances, but this would be considered a misclassification according to the reference data). Hence, forest degradation classification in regions with complex degradation histories need to take these factors into account.

The variety of vegetation types with different canopy textures that co-exist in the Amazon biome also poses a challenge to classify degraded forests. Canopy structure affects textural metrics from remotely sensed images [89] and its effects on the GLCM metrics must be assessed when classifying degraded forests for the entire Arc of Deforestation.

The construction of reference datasets is a recurrent challenge for any remote sensing classification. Making our approach operational would require both field and remote sensing expertise on the temporal and spatial patterns of forest degradation. Nonetheless, there is an increasing number of researchers and staff from environmental agencies that possess this expertise who could contribute to the generation of these data.

### Uncertainty in biomass estimation

We estimated ACD based on airborne lidar for different forest cover classes within each site. Following Chave, Condit [90], the estimated uncertainty of our lidar based ACD estimates accounts for uncertainty in the forest inventory estimates of ACD used for model calibration, uncertainty caused by the limited regions surveyed by both the airborne lidar and the ground-based measurements (representativeness), and the prediction error due to the ACD variance that cannot be explained by the fitted model [60, 91]. Similar to the findings of Chen, Vaglio Laurin [92], the model prediction uncertainty dominated this analysis. The representativeness uncertainty requires more attention. We used local data from the regions of Feliz Natal and Xingu as well as Saracá included in our ACD calibration, so we believe that representativeness is a minor issue for this study. However, for tropical forests in general, representativeness of data used for calibration of remote sensing studies is a serious concern. For most locations in tropical forests, the lack of ground-based forest inventory available for calibration could lead to large errors [93, 94].

We applied site-specific ACD distributions to represent the biomass in intact, burned, and logged forests at all three sites. Because we did not find any burned forests at the Saracá site as part of our calibration effort, we had no distribution of burned forest ACD based on lidar data from that site. We applied burned forest ACD distributions from Xingu and Feliz Natal for the Monte Carlo simulation for Saracá, which probably resulted in a bias towards low ACD given that the forests at Feliz Natal and Xingu had lower ACD compared to Saracá. Nonetheless, this bias had a limited effect on the site-level distribution because our probabilistic classification rarely estimated high probability of a grid cell being burned at Saracá site (about 1% of the study area, Fig. 4).

The propagation of uncertainty in forest degradation class together with uncertainty in ACD increased the uncertainty in the site-level carbon stock estimates. Unexpectedly, we found that consideration of both class and ACD uncertainty resulted in significantly lower mean carbon stock estimates in 2 out of 3 test sites (Fig. 7). Only the Xingu site showed similar ACD ranges with both uncertainty estimation methods, because it achieved the highest classification accuracy among the individual site models. We found no logging at Xingu in the reference data (Fig. 5) and the burned forest class showed the highest producer’s and user’s accuracy (Table 2). With minimal confusion between burned and logged classes at this site, there was no separation of the site level ACD estimates as we found in the other two sites.

The complex forest degradation history contributes to this unexpected effect. When both classification and ACD uncertainties are considered, mean ACD declined at two of our three sites (Saracá and Feliz Natal). This occurs because many grid cells classified as logged also have a finite probability of being classified as burned. Burned forests have lower ACD than logged forests so a mixture of burned and logged ACD in the Monte Carlo simulation resulted in a lower overall ACD estimate at two sites where a substantial portion of the landscape was logged. This effect did not occur in the Xingu site where, to our knowledge, there is no logging. The change in estimated ACD when forest classification is considered will not necessarily occur on all landscapes. Nonetheless, this observed change serves as an additional warning that it is important to consider the uncertainty in forest classification when estimating regional biomass in tropical forests.

Overlapping degradation events in space and time may also have affected the biomass estimates for the degradation classes. For instance, our choice to consider only the last disturbance may lead to biomass underestimation in logged areas that experienced earlier fires or increased biomass variability in sites that experienced multiple fires. This is more likely to happen in the Arc of Deforestation region where Feliz Natal and Xingu sites are located, and less likely to happen in more remote areas such as the Saraca-Taquera National Forest. Logging and forest fires can lead to changes in tree species composition. Following disturbance, we expect a shift from more shade tree tolerant species that generally have high wood density to more light demanding species that have low wood density [95]. Simulations suggest that this shift can lead to overall losses in tropical forest biomass [96]. While our lidar calibrations included intact, logged, and burned forests, they did not include many sites that had a long history of recovery from disturbance because those are rare in the Brazilian Amazon and difficult to document. Additional measurements of forest composition and structure are needed to resolve how compositional shifts can affect calibrations for airborne lidar based ACD estimates.

Our approach does not necessarily capture the biomass changes associated with fragmentation. Fragmentation leads to biomass loss on forest edges [49, 97–99]. Forest edges were included in our ACD distributions from earlier lidar data, so the effect of edges is implicit in our data. However, the loss of biomass from edges of intact forest is not contemplated in our analysis. We expect the effect of edges between degraded and intact forests to be smaller with larger edge effects in transitions from cleared areas to forests. Edge effects on biomass are also time varying [100] so we would need to consider time since disturbance to accurately quantify those effects. In particular, edge effects will be large in small forest fragments. Future studies incorporating explicit data on edge effects could better quantify the effects of fragmentation on forest biomass.

## Conclusions

We employed a classification model based on very-high resolution images to classify degraded forests in three Amazonian sites and obtained global accuracy of 0.86 on reserved test data not used in model training. Using airborne lidar data, we estimated biomass for intact, logged, and burned forests, and found that logged forests showed variable biomass changes due to logging, and that burned forests showed an average biomass loss of 35%. Subsequently, we used Monte Carlo simulations to assess how forest degradation classification affected biomass distributions in degraded forests. Increased uncertainty in forest degradation classification also leads to increases in uncertainty of biomass estimates. At two of three test sites, uncertainties in forest classification led not only to increased overall uncertainty but to significant shifts in mean ACD across these sites. These findings imply that this attribution needs to account for the uncertainty in forest degradation classification.

Commercial satellite VHR images have been available for over 20 years. Considering the robust market for this product, similar or more sophisticated images should be available at low cost for decades to come. The application of our method combining medium and VHR resolution images with airborne lidar data can provide critical information for national greenhouse gas inventories, because it allows the quantification, attribution, and estimation of uncertainties of carbon emissions associated with forest degradation through logging and fire.

## Data Availability

The airborne lidar data utilized in this study is available online at https://www.paisagenslidar.cnptia.embrapa.br/webgis/. The dataset supporting the conclusions of this article (reference data and GLCM metrics used as predictors, and the probability maps obtained as output of the probabilistic classification) is available in the NGEE-Tropics Data Archive repository, at the link https://doi.org/10.15486/ngt/1872685.
